# Intrahepatic Rupture of Acute Cholecystitis Complicated by Septic Portal Thrombosis

**DOI:** 10.7759/cureus.73865

**Published:** 2024-11-17

**Authors:** Mena Louis, Nathaniel Grabill, Baraa Mohamed, Firdous Khan, Joe Williams, Nelson A Royall

**Affiliations:** 1 General Surgery, Northeast Georgia Medical Center Gainesville, Gainesville, USA; 2 Surgery, Northeast Georgia Medical Center Gainesville, Gainesville, USA; 3 Gastroenterology, Augusta University Medical College of Georgia, Augusta, USA; 4 Gastroenterology and Hepatology, Philadelphia College of Osteopathic Medicine, Philadelphia, USA

**Keywords:** biliary fistula, biliary stent, cholangitis, cholecystitis, ercp, gallbladder, gallbladder rupture, liver abscess

## Abstract

Gallbladder rupture, though rare, is a serious complication often arising from choledocholithiasis and subsequent interventions such as endoscopic retrograde cholangiopancreatography (ERCP). In this case, the patient presented with acute choledocholithiasis and underwent ERCP with sphincterotomy and stone extraction, followed by placement of a fully covered metal stent in the common bile duct (CBD). While the use of covered stents is appropriate, it is important to note that these stents can obstruct the cystic duct orifice in patients with a gallbladder. This occurs in more than 33% of patients with a low cystic duct junction, leading to obstructive acute cholecystitis, as seen in patients with pancreatic ductal adenocarcinoma (PDAC) or distal cholangiocarcinoma who receive metal biliary stents. In this case, the patient developed a liver abscess following a gallbladder rupture, likely due to the stent obstructing the cystic duct. The liver abscess was managed with percutaneous drainage, and cultures grew *Streptococcus anginosus*, a common pathogen in hepatobiliary infections. The patient was treated with IV piperacillin-tazobactam, followed by oral amoxicillin-clavulanate for a 4-6 week course. Additionally, portal vein thrombosis, a known complication of severe infection, was identified and treated with anticoagulation. This case highlights the need for careful stent selection and possible prophylactic cholecystectomy in patients with a functioning gallbladder to prevent post-ERCP complications like cholecystitis and abscess formation. Early diagnosis, timely drainage, and appropriate antibiotic therapy are critical to managing such complex hepatobiliary conditions.

## Introduction

In managing choledocholithiasis, endoscopic retrograde cholangiopancreatography (ERCP) with stone extraction and stent placement is often the treatment of choice [1]. However, in patients with an intact gallbladder, the use of a fully covered metal stent presents a unique risk [2]. While effective at maintaining biliary drainage, these stents can inadvertently occlude the cystic duct orifice [3]. This leads to obstructive acute cholecystitis in a significant portion of patients, particularly those with a low cystic duct junction, making it a crucial consideration during stent selection and post-procedural care [4]. The incidence of acute cholecystitis following the placement of fully covered metal stents ranges from 3% to 12%, though progression to gallbladder rupture remains rare and is not well-documented.

Obstruction of the cystic duct orifice by a stent can trigger severe inflammation, bile stasis, and infection, as bile flow from the gallbladder is blocked [4]. Without appropriate intervention, this obstruction may progress to gallbladder rupture, biliary fistula formation, and the development of liver abscesses, as bile and bacteria infiltrate adjacent structures [5]. Early recognition of this complication, along with consideration for prophylactic cholecystectomy in certain high-risk patients, is essential to prevent serious outcomes and optimize recovery [6].

## Case presentation

A 30-year-old male presented with subacute progressive right upper quadrant abdominal pain, nausea, and bilious emesis. The patient denied any history of inflammatory bowel disease, alcohol use, sick exposures, or abdominal trauma/surgery. He had previous cocaine and methamphetamine abuse.

**Figure 1:**
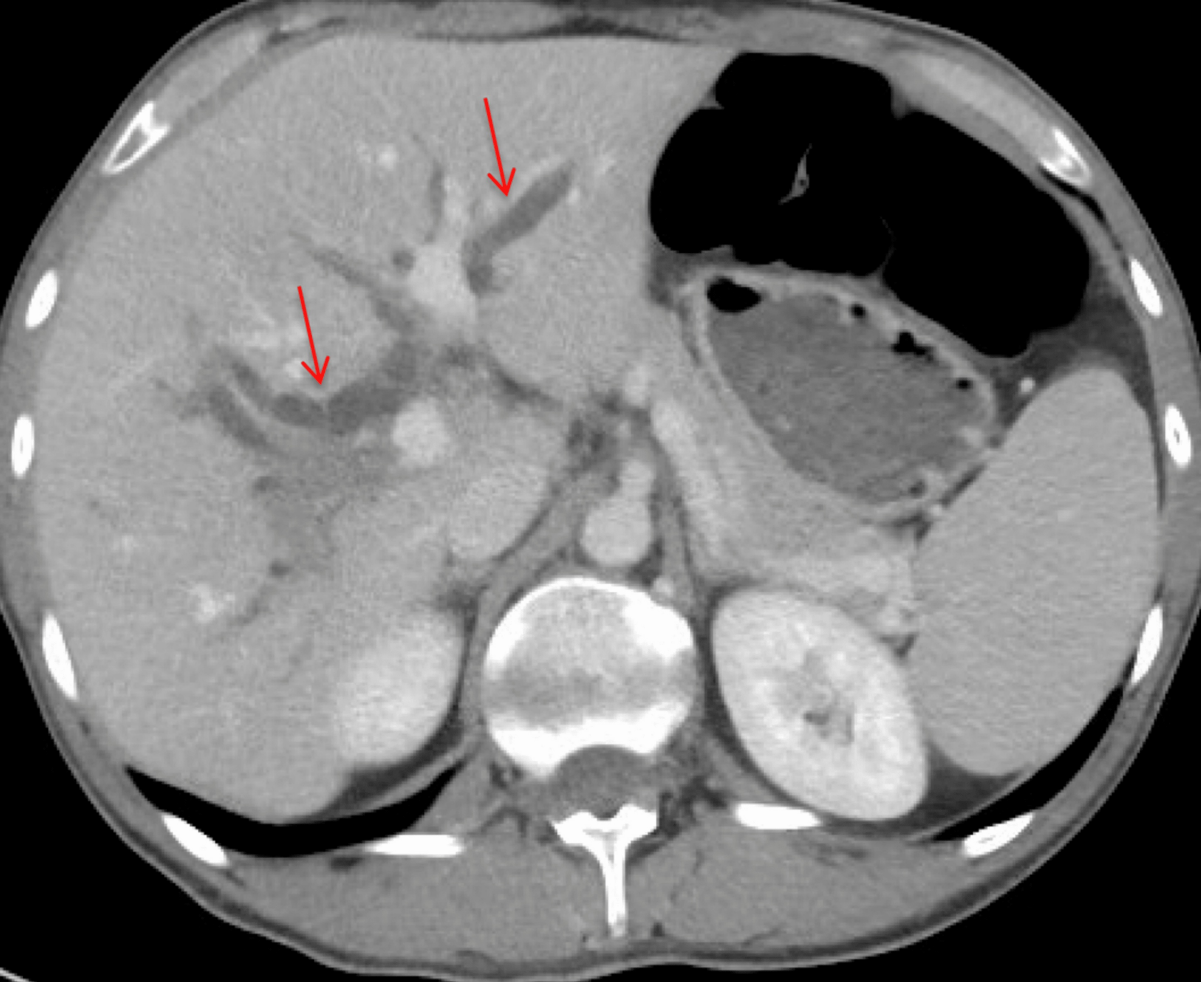
CT abdomen and pelvis with intravenous contrast (axial view) demonstrating intrahepatic and extrahepatic biliary ductal dilation (red arrows) with the CBD measuring up to 11 mm. CBD: common bile duct

On physical examination, the patient was afebrile with stable vital signs. Laboratory investigations are shown in Table 1. A CT scan of the abdomen and pelvis identified diffuse intra and extrahepatic biliary ductal dilation without obstructing stone or mass (Figure 1).

**Table 1:** Patient laboratory values on admission. AST: aspartate aminotransferase; ALT: alanine aminotransferase; H&H: hemoglobin and hematocrit; ALP: alkaline phosphatase; WBS: white blood cells; UA: urinalysis

Lab test	Result	Reference range
AST	138 U/L	10-40 U/L
ALT	218 U/L	7-56 U/L
Total bilirubin	9.0 mg/dL	0.1-1.2 mg/dL
Direct bilirubin	6.2 mg/dL	0-0.4 mg/dL
WBC	10.6 x 10^9^/L	4.5-11 x 10^9^/L
Lipase	25 U/L	0-160 U/L
UA (bilirubin)	Moderate	Negative
pH (UA)	8.0	4.5-8.0
Total protein (UA)	Trace	Negative
Total bilirubin (day 2)	7.29 mg/dL	0.1-1.2 mg/dL
AST (day 2)	36 U/L	10-40 U/L
ALT (day 2)	107 U/L	7-56 U/L
ALP (day 2)	237 U/L	44-147 U/L
WBC (day 2)	12.1 x 10^9^/L	4.5-11 x 10^9^/L
H&H	13.5/40.5	Hemoglobin: 13.5-17.5 g/dL, hematocrit: 41-50%
Platelets	342 x 10^9^/L	150-400 x 10^9^/L

**Figure 2:**
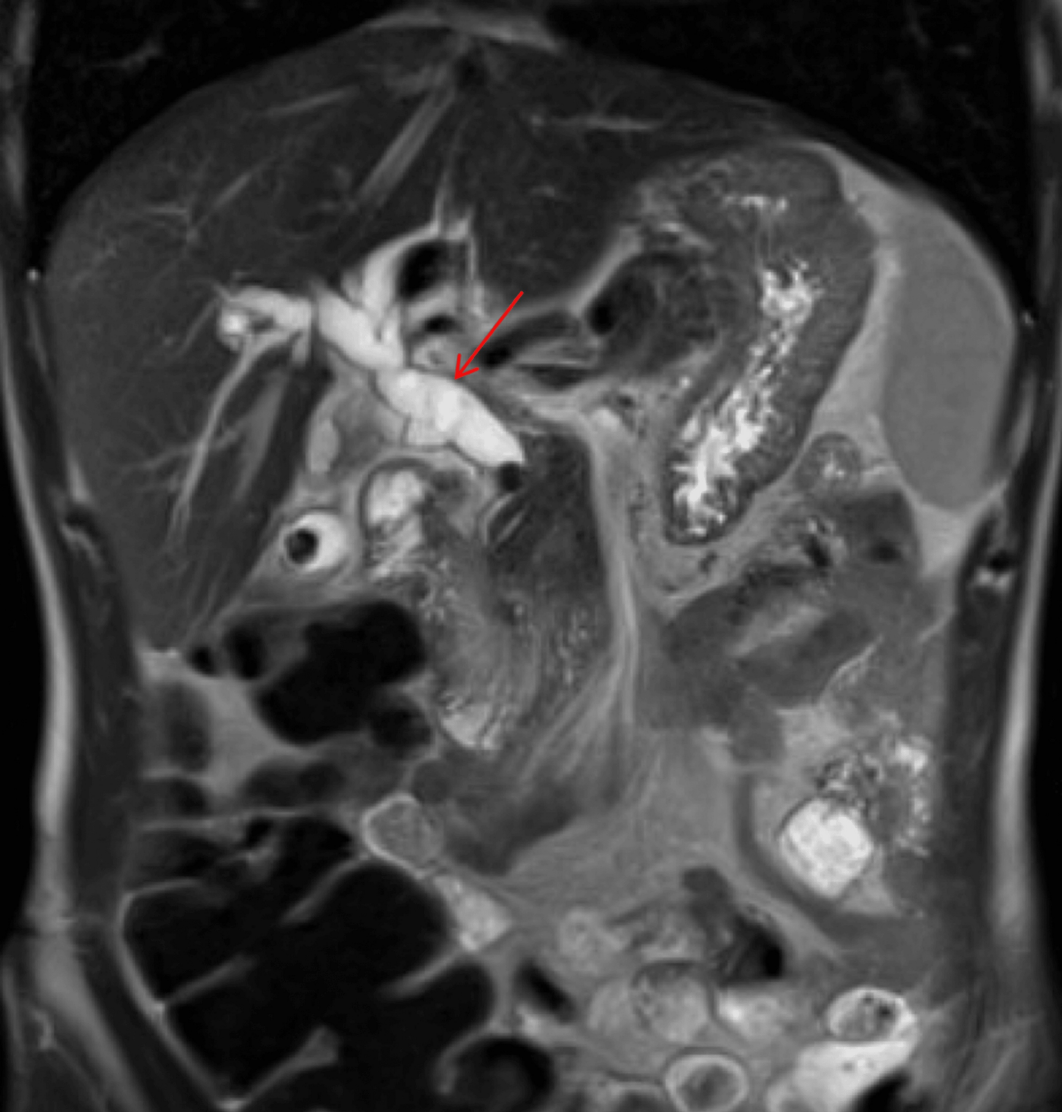
MRCP (coronal view) with marked intrahepatic and extrahepatic biliary ductal dilatation caused by a group of stones within the distal common bile duct (red arrow). MRCP: magnetic resonance cholangiopancreatography

**Figure 3:**
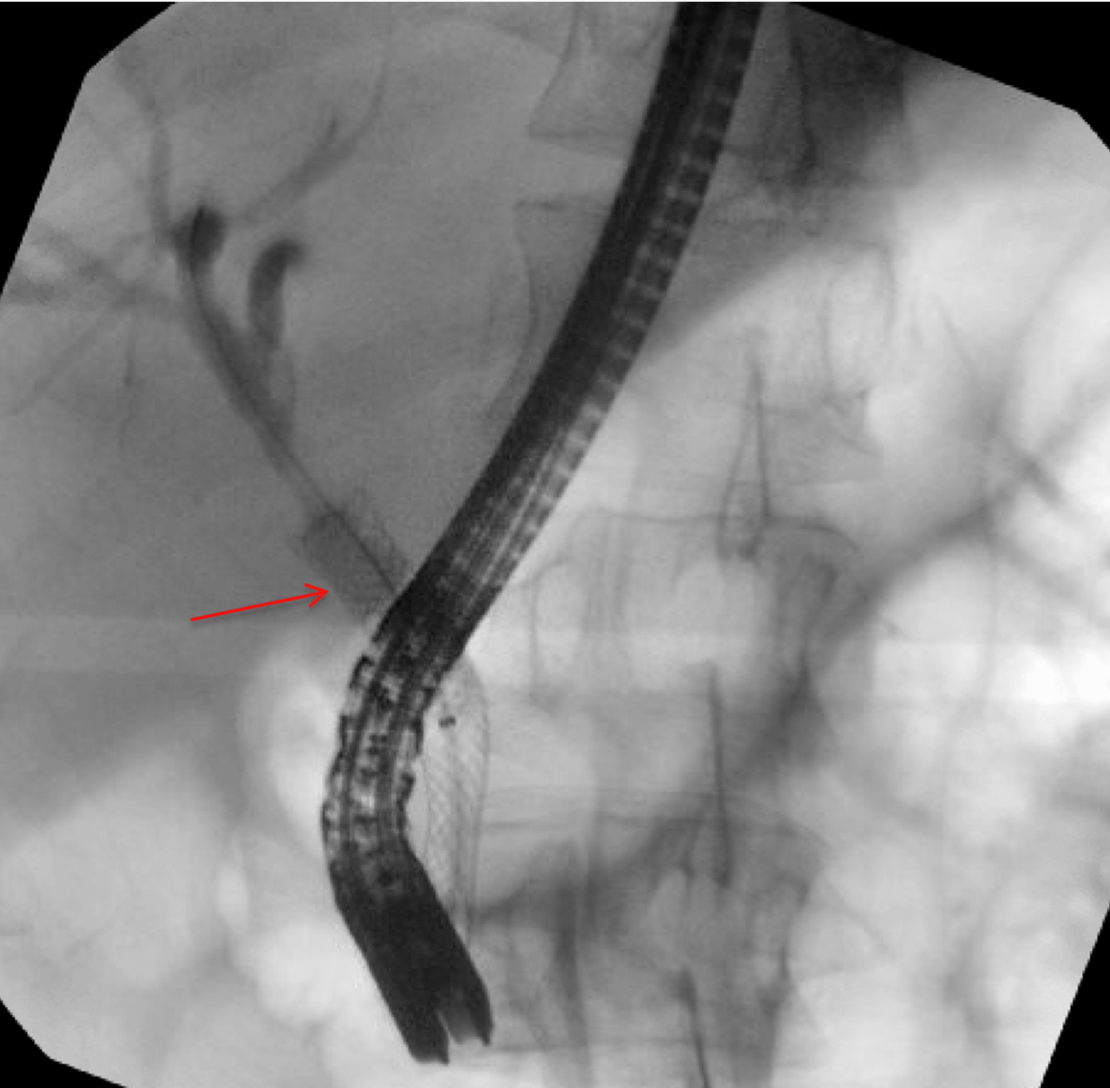
Fluoroscopic view of post-ERCP placement of a fully covered flexible metal biliary stent (red arrow) with good biliary drainage observed. Compared to the pre-procedure MRCP with a relatively low cystic duct junction, the proximal stent flange has occluded the level of the cystic duct junction. ERCP: endoscopic retrograde cholangiopancreatography; MRCP: magnetic resonance cholangiopancreatography

An MRCP demonstrated choledocholithiasis within the distal common bile duct (CBD) with significant intra and extrahepatic ductal dilation (Figure 2). He underwent an endoscopic retrograde cholangiopancreatography with sphincterotomy. Multiple stones were removed; however, due to concern for residual retained stones, a 10 French fully covered metal endobiliary stent was placed (Figure 3). A prophylactic plastic endopancreatic stent was also placed in the dorsal pancreatic duct due to incidental cannulation.

**Figure 4:**
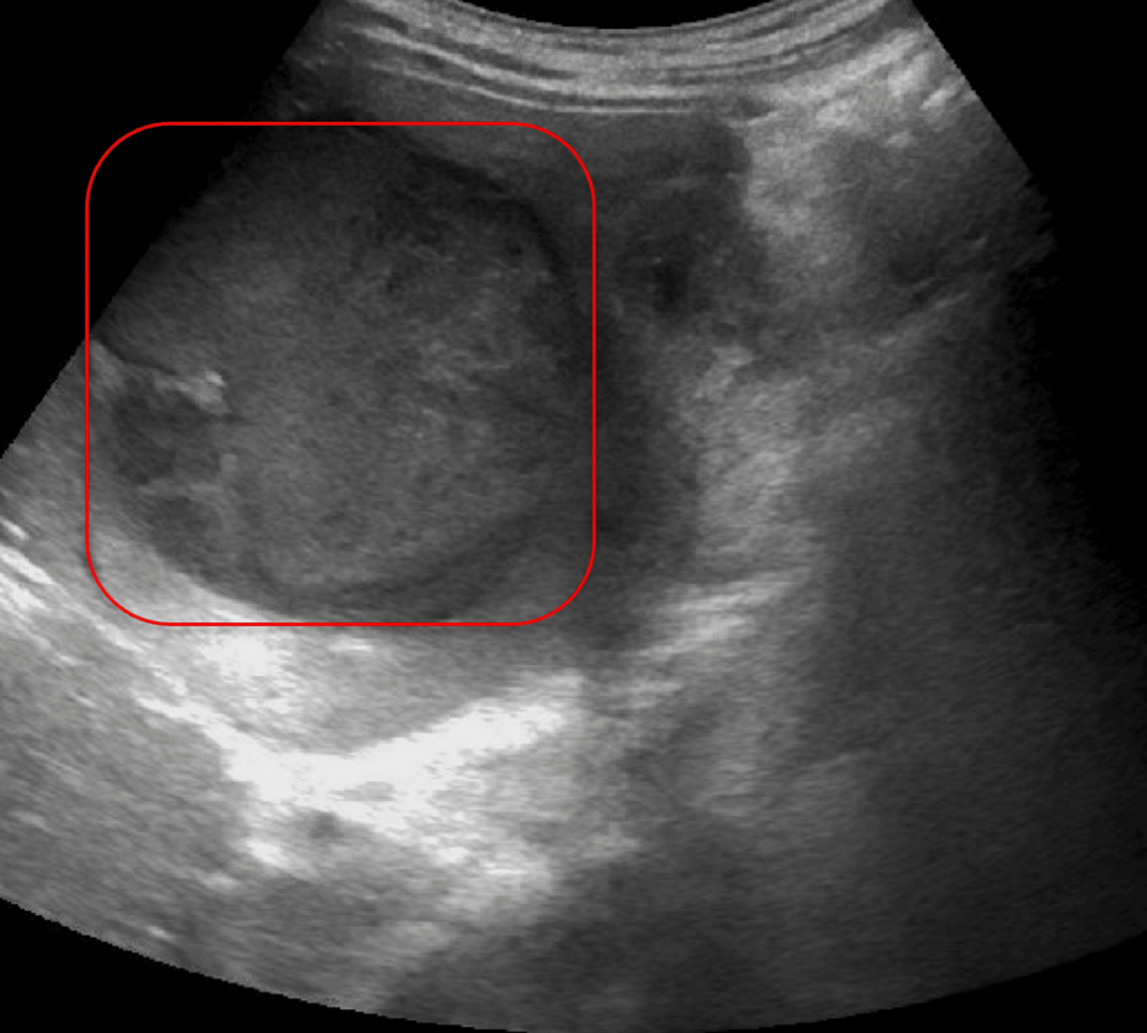
RUQ ultrasound with a complex fluid collection in the right hepatic lobe (red square). RUQ: right upper quadrant

**Figure 5:**
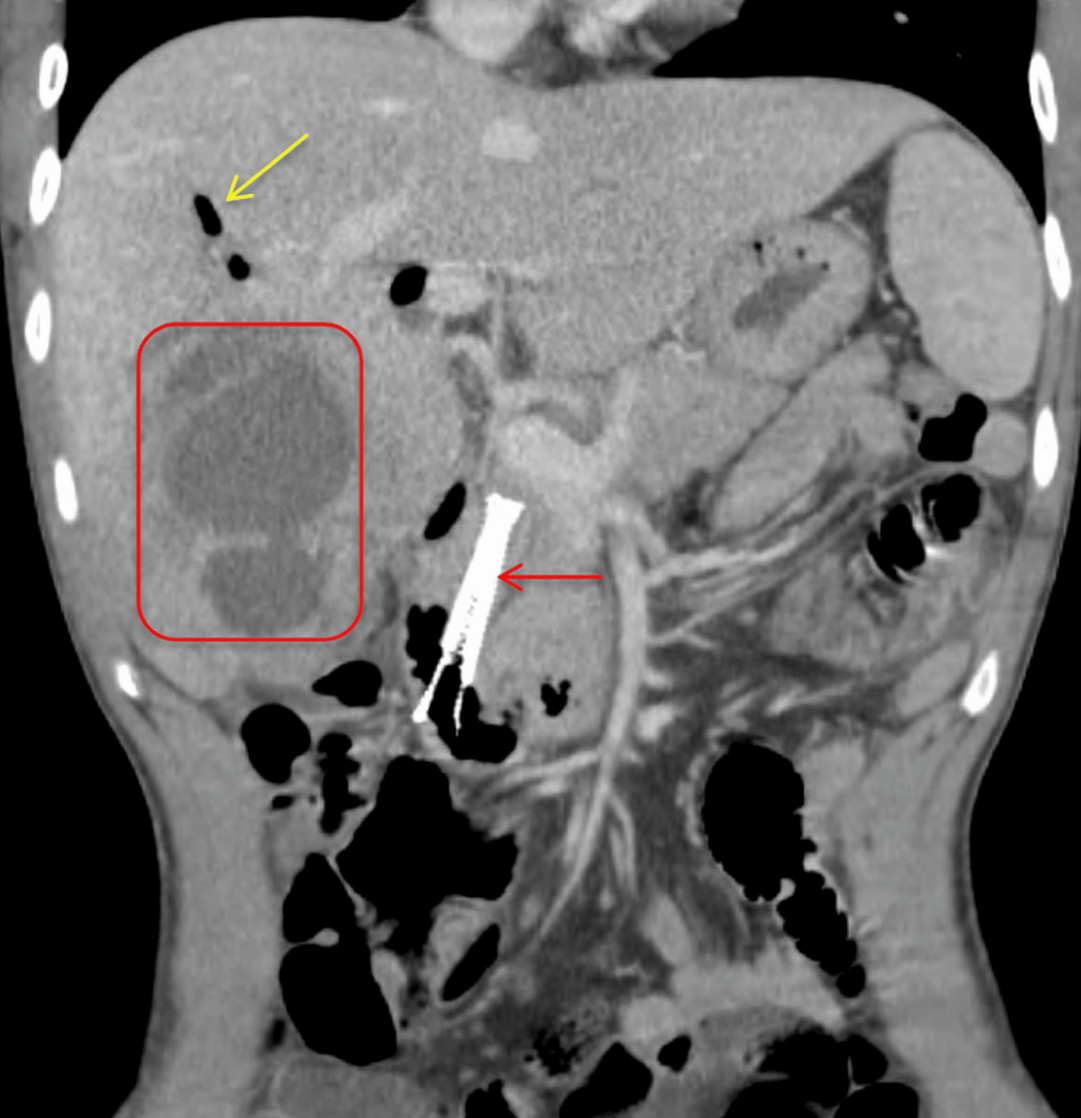
CT abdomen and pelvis with IV contrast (coronal view) demonstrating a peripheral enhancing fluid collection in the right hepatic lobe (red rectangle). Mildly distended intrahepatic biliary ducts are visible with air visible in the biliary tree (yellow arrow), likely related to the common bile duct stent extending into the duodenum (red arrow).

**Figure 6:**
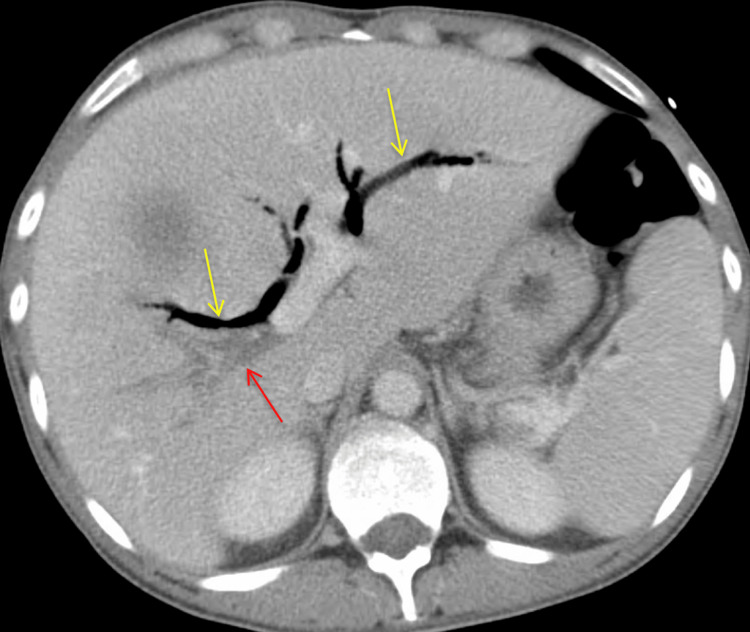
Additional view of CT abdomen and pelvis with IV contrast (axial view). The image shows the septic thrombosis of the right portal venous system (red arrow) with mildly distended intrahepatic biliary ducts and air in the biliary tree (yellow arrows).

Post-procedure, the patient developed improved jaundice and abdominal pain and was able to tolerate a regular diet with discharge home on the fifth post-procedure day. He re-presented three weeks later due to progressive cramping abdominal right upper quadrant (RUQ) pain, nausea, and bilious emesis. An abdominal ultrasound and CT demonstrated a large 8.5 cm liver abscess in the right hepatic lobe, with a posterior defect in the hepatic margin of the gallbladder wall (Figures 4-6). There was noted septic thrombosis of the right portal venous system. He underwent emergent CT-guided percutaneous transhepatic drainage of the liver abscess, with cultures demonstrating *Streptococcus anginosus*. He was initiated on empiric IV piperacillin-tazobactam, followed by a transition to a four-week course of oral amoxicillin-clavulanate 875-125 mg twice daily after sensitivity results were completed. He was initiated on therapeutic enoxaparin followed by conversion to oral apixaban for a planned six-month course for portal thrombosis. Repeat CT imaging demonstrated stable portal thrombus without extension or enlarging hepatic abscess. He was discharged home with planned interval endobiliary stent removal and delayed interval cholecystectomy. No complications or recurrent symptoms were observed during the patient's follow-up after discharge.

## Discussion

The use of a fully covered metal stent during ERCP is a common and effective solution for biliary drainage in cases of choledocholithiasis [7]. However, in patients with an intact gallbladder, the placement of such stents can introduce complications [8]. Fully-covered stents have a significant risk of obstructing the cystic duct orifice, particularly in patients with a low cystic duct junction [9]. This anatomical feature occurs in over 33% of cases, making it a critical factor to consider during stent placement [10,11]. When the cystic duct becomes obstructed, bile cannot drain from the gallbladder, leading to bile stasis and acute cholecystitis [12]. Without timely intervention, this can escalate into more serious complications, such as gallbladder rupture, as seen in this case [13]. While the use of fully covered metal stents in managing choledocholithiasis is common, cases like this one, where the stent placement led to cystic duct obstruction and subsequent gallbladder rupture, remain rare. Comparatively, most documented cases involving ERCP and stenting complications tend to present with acute cholecystitis rather than progressing to rupture and abscess formation [2].

Once the gallbladder ruptures, the potential for serious complications increases dramatically [14]. In this case, bile and bacteria leaked from the gallbladder into surrounding structures, forming a fistula between the gallbladder and the liver [13]. This direct communication allowed the spread of infection into the liver parenchyma, resulting in a large hepatic abscess [15]. Gallbladder ruptures are rare, but when they occur, they often lead to the development of liver abscesses, which are polymicrobial in nature [16]. *Streptococcus anginosus*, a frequent pathogen in biliary infections, was cultured from this patient’s abscess, consistent with the fistulous connection between the inflamed gallbladder and the liver [17,18]. Imaging, including ultrasound and CT, played a crucial role in diagnosing both the gallbladder rupture and the liver abscess, guiding the therapeutic approach [19].

ERCP with sphincterotomy and stone extraction remains the cornerstone of treatment for choledocholithiasis [1]. However, the decision to place a fully-covered metal stent in patients with an intact gallbladder requires careful consideration due to the risk of cystic duct occlusion [6]. In some cases, a prophylactic cholecystectomy should be considered, especially when the anatomy suggests a high likelihood of the stent covering the cystic duct [20]. In patients with pancreatic ductal adenocarcinoma (PDAC) or distal cholangiocarcinoma, the same risk exists when metal biliary stents are used, often resulting in fevers, chills, and right upper quadrant pain due to the blocked cystic duct [9]. Therefore, in patients receiving fully-covered metal stents, vigilant monitoring and consideration of surgical intervention are critical to prevent severe outcomes like obstructive cholecystitis or, as in this case, gallbladder rupture [21].

After the liver abscess was identified, prompt percutaneous drainage under CT guidance was performed to reduce the risk of further infection and sepsis [19]. Drainage remains the gold standard for managing large abscesses, especially when surgical resection is not feasible [15]. Coupled with targeted antibiotic therapy, drainage is critical for preventing the spread of infection and ensuring resolution [17]. In this case, the patient was initially treated with broad-spectrum antibiotics, including piperacillin-tazobactam, followed by a course of oral amoxicillin-clavulanate to cover the common biliary pathogens identified in the abscess culture. The prolonged antibiotic therapy helped prevent recurrence and allowed for a full recovery from the infection.

Complicating the course of this patient’s condition was the development of portal vein thrombosis, a recognized but serious complication of hepatobiliary infections [22]. Severe inflammation and infection within the liver can create a hypercoagulable state, increasing the risk of thrombosis in nearby vascular structures [23]. Portal vein thrombosis can lead to complications such as bowel ischemia or portal hypertension if left untreated [24]. In this case, therapeutic anticoagulation was initiated early to prevent these complications and promote resolution of the thrombosis.

Here, we illustrate the potential dangers associated with stent placement in patients with an intact gallbladder. The stent induced obstruction of the cystic duct, leading to gallbladder rupture. While ERCP remains an essential tool in managing choledocholithiasis, we must remain vigilant for post-procedure complications, particularly in patients receiving fully-covered stents. Early identification of complications such as liver abscesses and portal vein thrombosis, along with appropriate interventions like drainage and anticoagulation, are essential to ensure favorable outcomes in these complex cases. Alternative approaches, such as using partially covered or plastic stents, may reduce the risk of cystic duct obstruction in patients with an intact gallbladder. In high-risk cases, a prophylactic cholecystectomy could also be considered to prevent complications associated with stent-induced cystic duct occlusion.

## Conclusions

In patients with an intact gallbladder undergoing ERCP, fully covered metal stents should be used cautiously due to the risk of cystic duct obstruction, which can lead to complications like acute cholecystitis and, in rare cases, gallbladder rupture. Practitioners should consider alternative stenting options, such as partially covered or prophylactic cholecystectomy for high-risk patients. Prompt imaging, drainage, and targeted antibiotic therapy are essential for managing liver abscesses if they develop. Early recognition of complications and a multidisciplinary approach can significantly improve patient outcomes in such complex cases.
